# Short-Term Dynamics of North Sea Bacterioplankton-Dissolved Organic Matter Coherence on Molecular Level

**DOI:** 10.3389/fmicb.2016.00321

**Published:** 2016-03-15

**Authors:** Judith Lucas, Irina Koester, Antje Wichels, Jutta Niggemann, Thorsten Dittmar, Ulrich Callies, Karen H. Wiltshire, Gunnar Gerdts

**Affiliations:** ^1^Biological Station Helgoland, Shelf Sea Systems Ecology, Alfred-Wegener-Institute Helmholtz-Center for Polar and Marine ResearchHelgoland, Germany; ^2^Research Group for Marine Geochemistry (ICBM-MPI Bridging Group), Institute for Chemistry and Biology of the Marine Environment, Carl von Ossietzky University OldenburgOldenburg, Germany; ^3^Helmholtz-Zentrum Geesthacht Centre for Materials and Coastal Research, Institute of Coastal Research, Modelling for the Assessment of Coastal SystemsGeesthacht, Germany; ^4^Wattenmeerstation Sylt, Coastal Ecology, Alfred-Wegener-Institute Helmholtz-Center for Polar and Marine ResearchList/Sylt, Germany

**Keywords:** DOM, bacterioplankton community variation, short-term, FT-ICR-MS, 16S rRNA sequencing

## Abstract

Remineralization and transformation of dissolved organic matter (DOM) by marine microbes shape the DOM composition and thus, have large impact on global carbon and nutrient cycling. However, information on bacterioplankton-DOM interactions on a molecular level is limited. We examined the variation of bacterial community composition (BCC) at Helgoland Roads (North Sea) in relation to variation of molecular DOM composition and various environmental parameters on short-time scales. Surface water samples were taken daily over a period of 20 days. Bacterial community and molecular DOM composition were assessed via 16S rRNA gene tag sequencing and ultrahigh resolution Fourier-transform ion cyclotron resonance mass spectrometry (FT-ICR-MS), respectively. Environmental conditions were driven by a coastal water influx during the first half of the sampling period and the onset of a summer phytoplankton bloom toward the end of the sampling period. These phenomena led to a distinct grouping of bacterial communities and DOM composition which was particularly influenced by total dissolved nitrogen (TDN) concentration, temperature, and salinity, as revealed by distance-based linear regression analyses. Bacterioplankton-DOM interaction was demonstrated in strong correlations between specific bacterial taxa and particular DOM molecules, thus, suggesting potential specialization on particular substrates. We propose that a combination of high resolution techniques, as used in this study, may provide substantial information on substrate generalists and specialists and thus, contribute to prediction of BCC variation.

## Introduction

The global marine net primary production is estimated at 50 Gt C per year (Hedges, 1992). Part of this primary production is transferred to the marine dissolved organic matter (DOM) pool, making it one of the largest active carbon pool on earth (700 Gt), containing as much carbon as the Earth's atmospheric CO_2_ or all land plant biomass (Hedges, 1992). Bacterial consumption and remineralization of DOM via the microbial loop, transfers energy to higher trophic levels and thus, provides an important base for marine food webs (Azam et al., 1983; Azam, 1998). Between 30 and >90% of net primary production pass through the so-called labile dissolved organic carbon (DOC) fraction (Ducklow, 1999) and face rapid turnover by heterotrophic prokaryotes on a time scale of hours to days. Additionally, semi-labile DOC, which exhibits turnover times of months to years and can be followed as seasonal variability in DOC concentrations, provides support for the microbial loop (Hansell, 2013). Part of the DOC resists rapid bacterial degradation and as recalcitrant DOC, comprises a huge carbon pool of ~630 Gt, the largest part residing in the deep oceans. Since bacterial processing of DOM has large impact on global carbon and nutrient cycling, it is of great importance to understand how organic matter-bacteria interactions are controlled.

Different phylogenetic groups of bacteria tend to exploit different organic resources (e.g., Cottrell and Kirchman, 2000; Elifantz et al., 2005). This resource partitioning implies, that the DOM composition influences the bacterial community composition (BCC) and vice versa. Most studies that focus on DOM-bacterioplankton interactions are restricted to limited taxonomic resolution of microbial communities and selected compound classes of DOM. For instance, seasonal shifts of bulk DOC concentration and bacterial activity have been demonstrated over an annual cycle (Sintes et al., 2010). Venter et al. (2004) observed the tidal dynamics of DOC concentration and the bacterial community compositon. McCarren et al. (2010) examined the genomic and transcriptional response of microbial communities to addition of high molecular weight DOM in microcosms over the course of 1 day. However, only few studies observed interactions of bacterial communities and molecular DOM composition in marine systems (Osterholz et al., 2014, 2016; Medeiros et al., 2015; Seidel et al., 2015) and to our knowledge there is no study investigating the interactions of bacterial community variation and molecular DOM composition on high resolutions and short time-scales such as day to day variation.

This study examines short-term dynamics in bacterial community and DOM composition at Helgoland Roads (North Sea, German Bight) on a daily basis over a period of 20 days including the onset of the summer phytoplankton bloom. We hypothesize that changes in the BCC are closely linked to patterns in DOM composition and vice versa. We assessed the BCC via 16S rRNA gene tag sequencing and the DOM composition via electro-spray ionization (ESI) coupled with Fourier-transform ion cyclotron resonance mass spectrometry (FT-ICR-MS). Multiple regression analyses were used to identify environmental parameters that have fundamental impact on the bacterial community and DOM compositions.

## Materials and methods

### Site description and sampling

From August 6 to 26, 2012 a total of 17 surface water samples were collected daily at Helgoland Roads (North Sea, German Bight, 54°18.31 N, 7°88.97 E). Due to contamination and technical problems, samples from August 11, 15, 19, and 24 are not included in the data set. Environmental data including water level, water temperature, salinity, dissolved O_2_ and CO_2_ concentrations, turbidity, pH, SiO_2_, ${\text{PO}}_{4}^{3-}$, ${\text{NO}}_{2}^{-}$, ${\text{NO}}_{3}^{-}$, and chlorophyll *a* (Chl *a*) were obtained as part of the Helgoland Roads Time Series (Wiltshire et al., 2008). Data were measured with a Ferry Box system installed at the eastern pier at Helgoland. Water intake is at about 2–4 m depth, depending on tides. Data are accessible via the open database PANGAEA 2004 (http://www.pangaea.de).

Surface seawater was sampled always at 1 pm with a bucket, (initially thoroughly rinsed with surface seawater) from the pier at the inflow site of the Ferry Box. Samples were transferred to glass bottles (cleaned with acidified pH 2, ultrapure water and rinsed with sample water before use), transported to the lab immediately and further processed within 1 h at the Biological Station Helgoland.

### 16S rRNA gene tag sequencing of bacterial communities

Five hundred milliliters of surface seawater were vacuum filtered through 0.22 μm polycarbonate filters (GTTP, Ø 47 mm, Merck Millipore, USA) using bottletops cleaned with pH2, ultrapure water, and rinsed with sample water before use, to obtain bacterial biomass. Filters were stored at −20°C and further processed within 4 weeks. DNA extraction was carried out as described in Sapp et al. (2007). Briefly, lysozyme and sodium dodecyl sulfate were used for cell lysis followed by extraction with phenol-chloroform-isoamyl alcohol (25:24:1) and precipitation with isopropanol. DNA concentration per sample and purity were determined photometrically using a Tecan Infinite©200, NanoQuant microplate reader (Tecan, Switzerland).

16S rRNA gene tag sequencing was performed at LGC Genomics GmbH (Berlin, Germany). Community DNA samples were sent to LGC in a 96-well plate for generation of 16S V4 rRNA amplicon libraries for Illumina sequencing. Community DNA was amplified utilizing amplification primers targeting the V4 region of the 16S rRNA gene using 515F (5′ GTGCCAGCMGCCGCGGTAA 3′) and 806R (5′ GGACTACHVGGGTWTCTAAT 3′) (Caporaso et al., 2011). Primers also contained the Illumina sequencing adapter sequence and a unique barcode index. Sequencing was done on an Illumina MySeq platform using 2 × 250 bp chemistry. Raw paired-end reads were merged using the FLASh 1.2.4 software (http://ccb.jhu.edu/software/FLASH/) and processed through the SILVAngs pipeline (Quast et al., 2013). Sequences were de-replicated at 100% identity and then clustered within each individual sample at 98% similarity. Representative sequences from operational taxonomic unit clusters (OTUs) were classified up to genus level against the SILVA v119 database using BLAST as described by (Ionescu et al., 2012). Genus-level classifications were used in the final abundance matrix for downstream analyses. Each classification contained the sum of all sequences represented by OTUs with the same taxonomic path. For the purposes of this study we were not interested in diversity calculated at the level of 98% clustered OTUs but rather used BLAST identities as our OTU. From this point on, we define these taxa as OTUs for simplicity. Eukaryotic, chloroplast and mitochondria-derived OTUs were removed from the resulting OTU matrix. Only OTUs with an average relative abundance ≥0.1% were considered for further analysis.

Sequence data were deposited in the Sequence Read Archive (SRA) of the National Center for Biotechnology Information (NCBI) under accession number SRP058371.

### Dissolved organic matter (DOM)

For DOM extraction, 2 l of each sample were filtered through 2 and 0.7 μm glass fiber filters (GMF and GF/F, Whatman, United Kingdom, combusted at 400°C, 4 h), acidified to pH 2 (HCl 32% p.a., Carl Roth, Germany), and stored at 4°C in the dark. Aliquots of the acidified 0.7 μm filtrate were sampled for quantification of DOC and total dissolved nitrogen (TDN). DOC and TDN concentrations were analyzed by high-temperature catalytic combustion using a TOC-VCPH/CPN Total Organic Carbon Analyzer equipped with an ASI-V autosampler and a TNM-1 module (Shimadzu, Japan). Prior to analysis, the acidified samples were purged with synthetic air to remove dissolved inorganic carbon. L-arginine solutions ranging from 5 to 500 μmol C l^−1^ and 6.6 to 333.3 μmol N l^−1^, respectively, were used for calibration and Deep Atlantic Seawater reference material (DSR, D. A. Hansell, University of Miami, Florida, USA) was measured during each run to control for instrumental precision and accuracy. Samples were measured in duplicates, average deviation of duplicate analysis was 4.4% for DOC and 1.6% for TDN.

DOM was extracted using modified styrene divinyl benzene polymer cartridges (PPL, Agilent, USA) as described in Dittmar et al. (2008). Cartridges were rinsed with two cartridge volumes of pH 2 ultrapure water to remove remaining salts, dried with inert pure argon gas and eluted with 6 ml methanol (ULC/MS grade, Biosolve, Netherland) into amber vials. The extract volume was determined by weight. Hundred microliters of the methanol extracts were evaporated overnight and re-dissolved in 10 ml ultrapure water at pH 2 for DOC analysis. The extraction efficiency was calculated as percentage DOC amount of the extract on the DOC amount of the original sample.

Mass spectra were obtained using a 15 T Solarix FT-ICR-MS (Bruker Daltonics, USA) equipped with an electrospray ionization source (Bruker Apollo II) applied in negative mode. Methanol extracts were diluted in a 1:1 ratio with ultrapure water to a final concentration of 20 mg C l^−1^. A total of 500 scans were accumulated per run and mass spectra were evaluated in the range from 150 to 2000 Da. Mass spectra were calibrated with an internal calibration list of known molecular formulae mass peaks (Bruker Daltonics Data Analysis 4.0 SP 3 software package). Mass to charge ratios, peak intensities, and resolutions were exported and molecular formulae were assigned to the detected mass peaks with a minimum signal-to-noise ratio of 4, according to Koch et al. (2007). Masses were kept for further data analysis when detected in more than two samples. Masses present in less than 20% of the samples were allowed if the S/N ratio was >20 in at least one sample. Additionally, formulae were deleted that contained following combinations of heteroatoms: NSP, N_2_S, N_3_S, N_4_S, N_2_P, N_3_P, N_4_P, NS_2_, N_2_S_2_, N_3_S_2_, N_4_S_2_, PS_2_. Remaining double assignments were removed. Peak intensities were normalized to the sum of peak intensities of all masses and considered as quantitative measure (relative abundances) of the respective DOM formulae. The ability of FT-ICR-MS peaks to be quantitative and reproducible has been evidenced by numerous studies (e.g., Riedel and Dittmar, 2014; Osterholz et al., 2015; Seidel et al., 2015; Zark et al., 2015). Masses which are listed as known contaminations including their homologous series and all isotopologs were removed. For each assigned formula the double bond equivalents [DBE = 1 + ½(2C−H + N + P)] as a measure for the degree of unsaturation (Koch and Dittmar, 2006) and the modified aromaticity index [AI_mod_ = (1 + C−½O−S−½H)/(C−½O−S−N−P)] were calculated to assess the presence and abundance of aromatic structures (Koch and Dittmar, 2006). Based on elemental ratios, AI_mod_ and heteroatom contents, molecular formulae can be categorized into compound groups (Seidel et al., 2014).

### Statistical analysis

Principal coordinates analyses (PCoA) were accomplished to reveal patterns in environmental parameters, BCC and DOM composition. Environmental variables were log transformed and normalized prior to analyses. PCoA for environmental parameters was carried out using Euclidean distances. Patterns in BCC were revealed by conducting PCoA of OTU read numbers using Hellinger distance (Legendre and Legendre, 1998), which uses square root transformed relative abundances for distance calculation. Patterns in DOM composition were observed based on Bray-Curtis distances, generated from square root transformed mass spectrometric data.

Samples were grouped via non-hierarchical group-average linkage clustering implemented in the non-parametric *k-R clustering* approach of the Primer v.7 software package (PRIMER-E, UK). In this approach, the classic idea of *k-means clustering*, which seeks to minimize within-group sums of squares about *k* group centroids, is generalized to non-parametric *k-R clustering* which analogously maximizes ANOSIM R and thus, allows the application of any resemblance measure desired. Based on the PCoA patterns the desired number of groups was specified as per authors discretion *a priori* to *k* = 3 for environmental data and *k* = 2 for 16S rRNA tag sequencing and DOM data. An iterative search then attempts to divide the samples into k groups in such a way that samples with greatest similarities (defined as the average of the pairwise similarities between a sample and all members of the same group) fall into one group. Significance of groups was confirmed using permutational multivariate analysis of variance (PERMANOVA) with fixed factors and 999 permutations at a significance level of *p* < 0.05 (see Table S3). Analysis of variance (ANOVA) was applied at a significance level of *p* < 0.05 using Statistica 11 (StatSoft, USA), to test for significant difference of single environmental parameters between groups of samples.

The linear discriminant analysis effect size method (LEfSe; Segata et al., 2011) was used to determine particular bacterial taxa and DOM molecules which were most likely to explain differences between the two groups of samples. LEfSe uses the non-parametric factorial Kruskal-Wallis sum-rank test to detect features (OTUs or DOM molecules respectively) with significant differential abundance with respect to the groups of interest. Linear discriminant analysis (LDA) is then used to rank features according to their relative difference (effect size) among groups. Kruskal–Wallis tests were done on a significance level of *p* < 0.05. The threshold on the logarithmic LDA score for discriminative features was set at 2. An implementation of LEfSe including a convenient graphical interface incorporated in the Galaxy framework (Giardine et al., 2005; Blankenberg et al., 2010; Goecks et al., 2010) is provided online at http://huttenhower.sph.harvard.edu/lefse/.

Correlations between all environmental parameters were determined using Spearman rank order correlation (Statistica 11, StatSoft, USA) to reveal multicollinearities. Based on these correlations, environmental parameters were selected for multiple regression analysis to unravel their relationship with BCC and DOM composition. Multiple regression analyses were performed using distance-based linear modeling (DistLM). DistLM models were build using stepwise selection, adjusted *R*^2^ and applying 999 permutations at a significance level of *p* < 0.05. Due to observed multicollinearity, the variables pH, turbidity and CO_2_ were excluded from the analysis (see Results part for further explanation). Results were visualized via distance-based redundancy analysis (dbRDA). All multivariate analyses were performed using the Primer v.7 software package (PRIMER-E, UK). To further unravel the relationship of DOM molecules with specific environmental parameters, correlations of DOM molecules with salinity, temperature, and DOC were calculated using Pearson product-moment correlation (Statistica 11, StatSoft, USA).

To investigate the relationship between specific OTUs, DOM compounds and environmental parameters, pairwise correlations were calculated with R (R Development Core Team, 2014) using Pearson product-moment correlation at a significance level of *p* < 0.05. When considering several hypotheses in the same test the problem of multiple statistical inference arises (Holm, 1979). If one accounts for this family-wise error rate, e.g., via the Holm-Bonferroni correction (Holm, 1979), few of the apparent correlations would remain statistically significant. We compared raw data of OTU relative abundances and molecular formulae intensities to demonstrate that the observed correlations are plausible and consistent, and do not occur in a random fashion (Figure S2). High correlations (*r* ≤ −0.9 or ≥ 0.9) were visualized in a network constructed using Cytoscape version 3.2.0 (Shannon et al., 2003).

## Results

### Oceanographic conditions at sampling site

**Figure 1:**
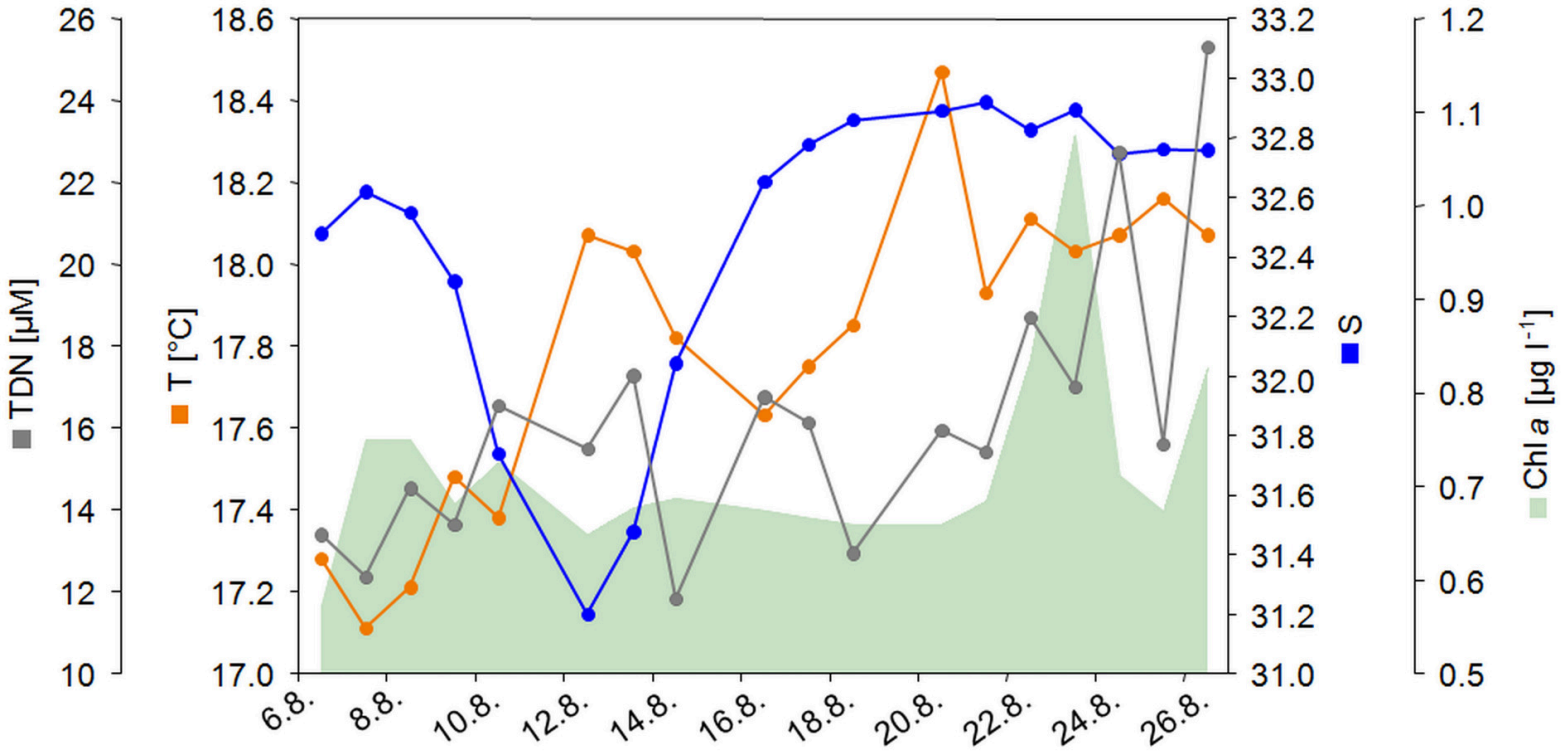
**Salinity (S), temperature (T), total dissolved nitrogen (TDN), and Chlorophyll ***a*** (Chl ***a***) concentration during the sampling period from August 6 to August 26, 2012**.

Concurrent with water sampling, various physico-chemical parameters and nutrient concentrations were recorded (Table S1). Most striking was the high variation in salinity during the sampling period (Figure 1). During the first week of sampling, salinity decreased from 32.6 to 31.2 on August 12 2012, followed by an increase to 32.9 in the following week. Additionally, TDN concentration and temperature increased over the sampling period (Figure 1). The Chl *a* concentration increased toward the end of the sampling period, indicating the onset of a summer phytoplankton bloom. Spearman rank order correlations revealed strong significant multicollinearity (*p* < 0.5, *r* > 0.6, Graham, 2003) among turbidity and Chl *a* (*R* = 0.890), salinity and pH (*R* = −0.799), salinity and DOC (*R* = −0.600), O_2_ and CO_2_ (*R* = −0.807), temperature and ${\text{NO}}_{2}^{-}$ (*R* = 0.645), ${\text{NO}}_{3}^{-}$ and SiO_2_ (*R* = 0.607), and depth and pH (*R* = −0.620; Table S2). The power to detect a significant effect of a predictor on a response variable decreases nonlinearly with increasing multicollinearity (Graham, 2003). Therefore, we decided to drop collinear variables from further analysis, knowing that this might result in a substantial loss of overall explanatory power. Based on previous studies that uncovered Chl *a* (as proxy for phytoplankton abundance) and salinity as important driving factors for bacterial community dynamics (Fortunato et al., 2012; Lucas et al., 2015) we decided to treat pH, turbidity and CO_2_ as functionally less important and excluded those variables from all further analyses.

**Figure 2:**
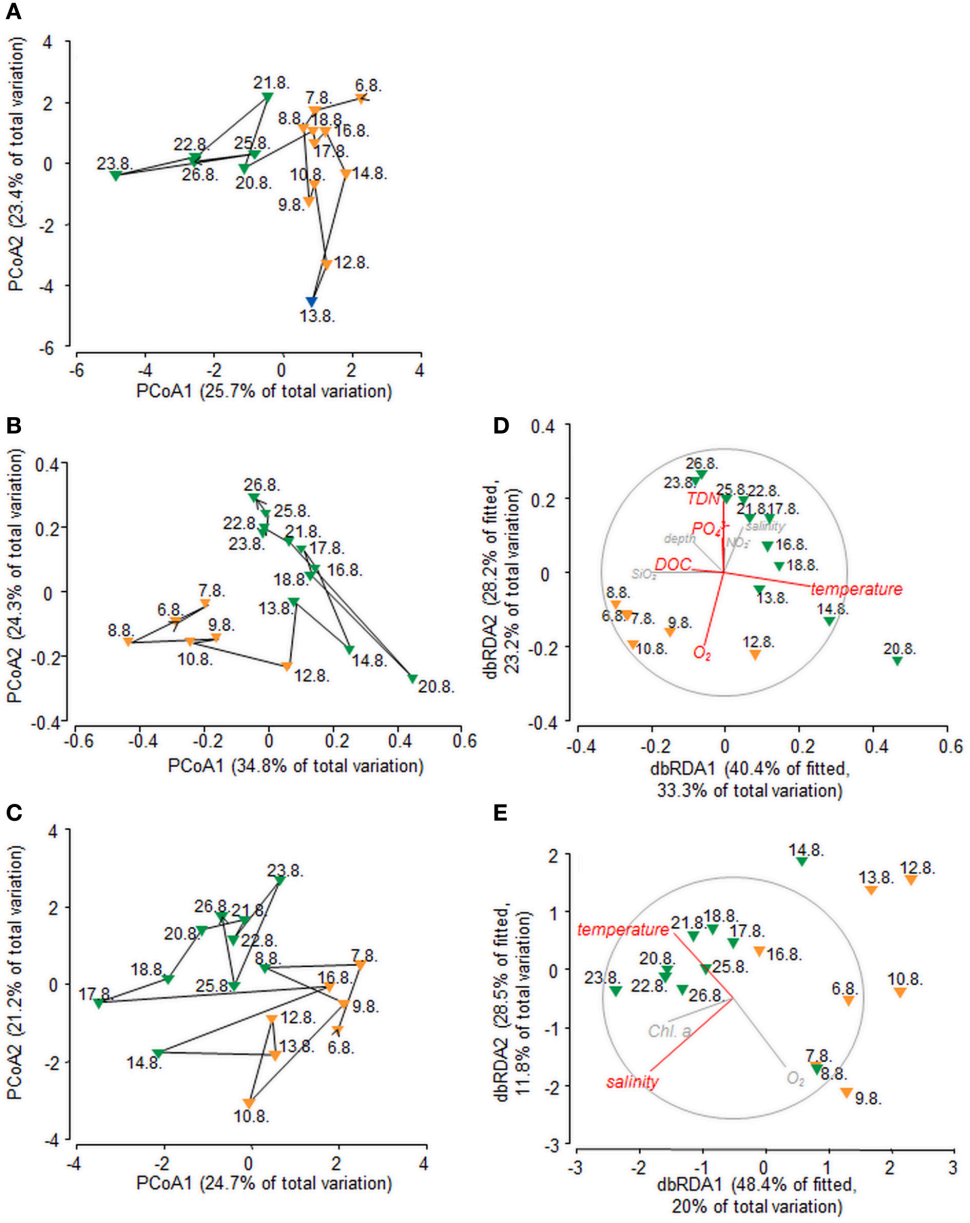
**Principal coordinates analyses (PCoA) of (A) environmental variables based on Euclidean distance, (B) bacterial communities based on Hellinger distance, and (C) molecular DOM composition based on Bray-Curtis similarity**. Distance-based redundancy analysis (dbRDA) of **(D)** bacterial communities and **(E)** DOM composition, both based on Bray-Curtis similarities. Environmental parameters explaining the variation significantly (*p* < 0.05) are depicted in red, non–significant parameters are depicted in gray. Color code refers to group formation according to *k*-R *Clustering*. Orange, group A; Green, group B; Blue, group C.

PCoA of environmental data suggested that samples might cluster in three groups (Figure 2A), reflecting pronounced changes in environmental conditions during the sampling period. Non-hierarchical *k-R clustering* results, however, revealed that the third group was built by a single sample (13.8.). Thus, a separation into two groups (group A and B) appeared more reasonable and the sample 13.8. was added to group A during all following analyses. ANOVA confirmed significant (*p* < 0.05) differences between both groups for TDN, temperature, salinity and Chl *a* (Table S3). Group A is characterized by lower average temperature (17.6°C), salinity (32.25), TDN (14.61 μM), and Chl *a* (0.68 μg l^−1^) concentrations compared to group B where the average values were 18.13°C, 32.84, 17.98 μM TDN, and 0.79 μg l^−1^ Chl *a*.

### Bacterial community composition, variation, and relation to environmental parameters

A total of 1,720,615 high quality sequences were obtained, clustering into 98 different taxonomically assigned OTUs (Table S4). During the sampling period the community was mainly composed of *Proteobacteria* (52.8%), *Bacteroidetes* (30.8%), and *Actinobacteria* (4.8%). On class level, *Flavobacteriia* was the predominant group (27.3%), closely followed by *Alphaproteobacteria* (26.8%) and *Gammaproteobacteria* (21.8%). Another highly abundant class was *Acidimicrobiia* (6.6%).

Prevailing OTUs within the *Flavobacteriia* were the NS5 marine group, *Tenacibaculum* and a *Cryomorphaceae* related cluster (Figure S1). *Alphaproteobacteria* were dominated by OTUs affiliated with the *Roseobacter* clade (*Candidatus Planktomarina*, NAC11-7 lineage, OCT lineage and *Sulfitobacter*). The prominent G*ammaprotoeobacteria* were OM60(NOR5) clade, *Oceanospirillales* related clone ZD0405 and SAR86 clade. Other OTUs with high relative abundances were Candidatus *Actinomarina*, Marine group II (*Euryarchaeota*).

**Figure 3:**
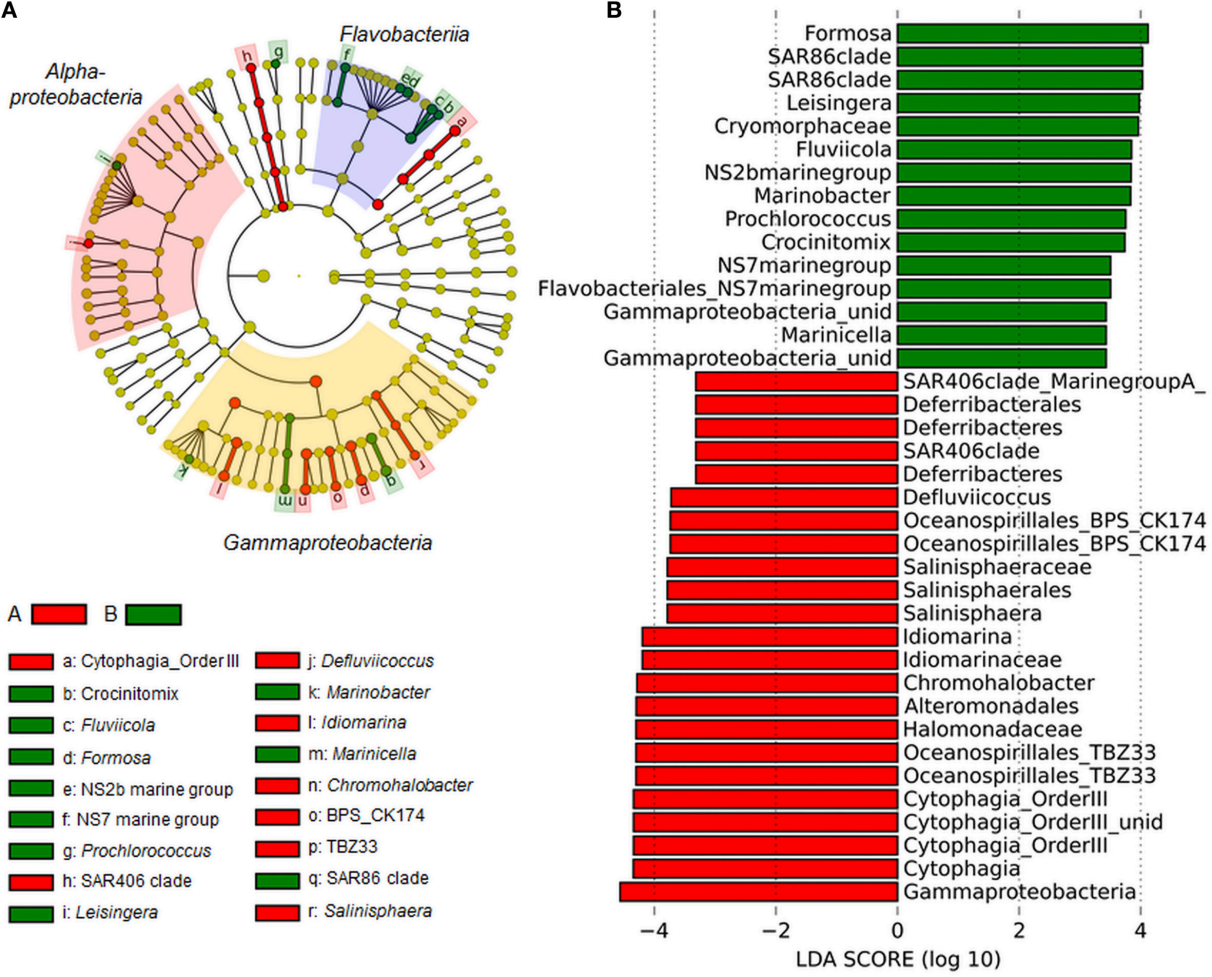
**Linear discriminant effect size analysis (LEfSe) results on bacterioplankton communities. (A)** Taxonomic representation of statistically consistent differences between group A and B. Differences are represented in the color of the group, where the OTU is most abundant. Colored areas mark the most prominent bacterial classes found during this study. Red, *Alphaproteobacteria*; Yellow, *Gammaproteobacteria*; Blue, *Flavobacteriia*. **(B)** Histogram of linear discriminant analysis (LDA) scores computed for OTUs, differently abundant in group A and B. LDA scores can be interpreted as the degree of consistent difference in relative abundance between the two groups. The histogram thus identifies which OTUs among all those detected as statistically different explain the greatest difference between group A and B.

PCoA and non-hierarchical clustering of bacterial community tag data revealed a separation of samples into two groups (Figure 2B). DistLM analysis suggested that temperature, TDN, O_2,_${\text{PO}}_{4}^{3-}$ and DOC significantly influenced this group formation (Figure 2D, Table S5). To determine which bacterial taxa were most likely contributing to the differences in community composition between the two groups, linear discriminant effect size analysis (LEfSe) was performed (Figures 3A,B). In general, *Alpha- and Gammaproteobacteria* were dominating group A (25.2 and 26.8%). *Gammaproteobacteria* decreased in relative abundance in group B (19.1%), whereas *Alphaproteobacteria* increased slightly (27.6%). Most interestingly, *Flavobacteriia* reached a relative abundance of 30.1% in group B and became the dominating class. In particular the *Gammaproteobacteria* OTUs TBZ33 and BPS-CK174 (*Oceanospirillales*)*, Chromohalobacter, Idiomarina, Salinisphaera, Marinobacter, Marinicella*, and SAR86 clade, the *Flavobacteriia* related OTUs *Formosa, Fluviicola, Crocinitomix*, NS2b, and NS7 marine group, and the *Alphaproteobacteria* related OTUs *Defluviicoccus* and *Leisingera* contributed most to the differences between the groups A and B (Figures 3A,B).

### DOM composition, variation, and relation to environmental parameters

The average solid phase extraction efficiency was 44% (± 3.3%). A total of 4039 molecular formulae were assigned, ranging between 3842 and 3947 formulae per sample (average of all samples: 3892). The identified peaks covered a mass range from 159 to 809 Da with weighted average masses per sample between 370.2 and 385.4 Da (average of all samples: 377.1 Da).

**Figure 4:**
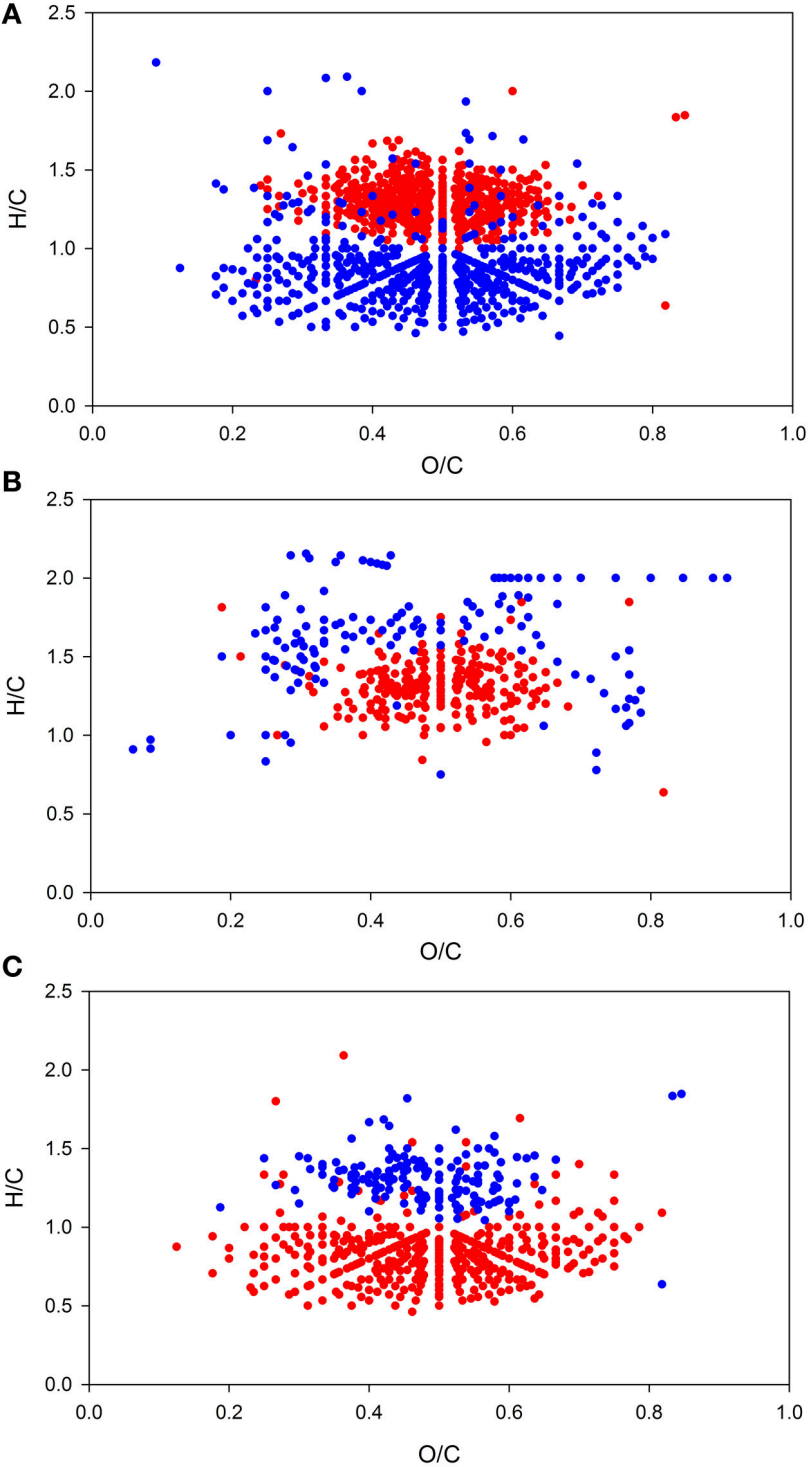
**Van Krevelen plots of DOM molecular formulae with relative intensity correlating significantly (***p*** < 0.05) with salinity (A), temperature (B), and dissolved organic carbon concentration (DOC) (C)**. Molecular formulae showing positive correlations are depicted in red; formulae with negative correlations are shown in blue.

As for BCC and for environmental parameters, PCoA and non-hierarchical clustering revealed a separation of samples into two groups (Figure 2C). DistLM analysis identified salinity and temperature as main influencing factors (Figure 2E, Table S5). However, temperature exhibited a significant (*p* < 0.05) correlation with TDN (*R* = 0.508) and ${\text{NO}}_{2}^{-}$ (*R* = 0.645) and salinity exhibited a significant correlation with DOC (*R* = −0.6) and O_2_ (*R* = −0.556), thus, there might be a shared contribution to the explanation of variation in DOM composition. Elemental ratios of assigned molecular formulae provide information on molecular characteristics, which can be visualized in van Krevelen diagrams (Kim et al., 2003). Van Krevelen plots of all molecules that were significantly correlated (*p* < 0.05) with either salinity, temperature, or DOC revealed the nature of these relationships in more detail (Figure 4). Molecules that were positively correlated with salinity had higher H/C ratios and were clearly distinguished from molecules that were negatively correlated with salinity and showed lower H/C ratios (Figure 4A). Molecules that were positively correlated with temperature formed a dense cluster in the center of the van Krevelen diagram, whereas molecules negatively correlated with temperature were more scattered, showed higher H/C ratios and covered a broader range of O/C ratios (Figure 4B). The distribution of H/C and O/C ratios of molecules significantly correlated (*p* < 0.5) with DOC is depicted in Figure 4C. Molecules that were positively correlated with DOC showed low H/C ratios, while negatively correlated molecules exhibited higher H/C ratios.

**Figure 5:**
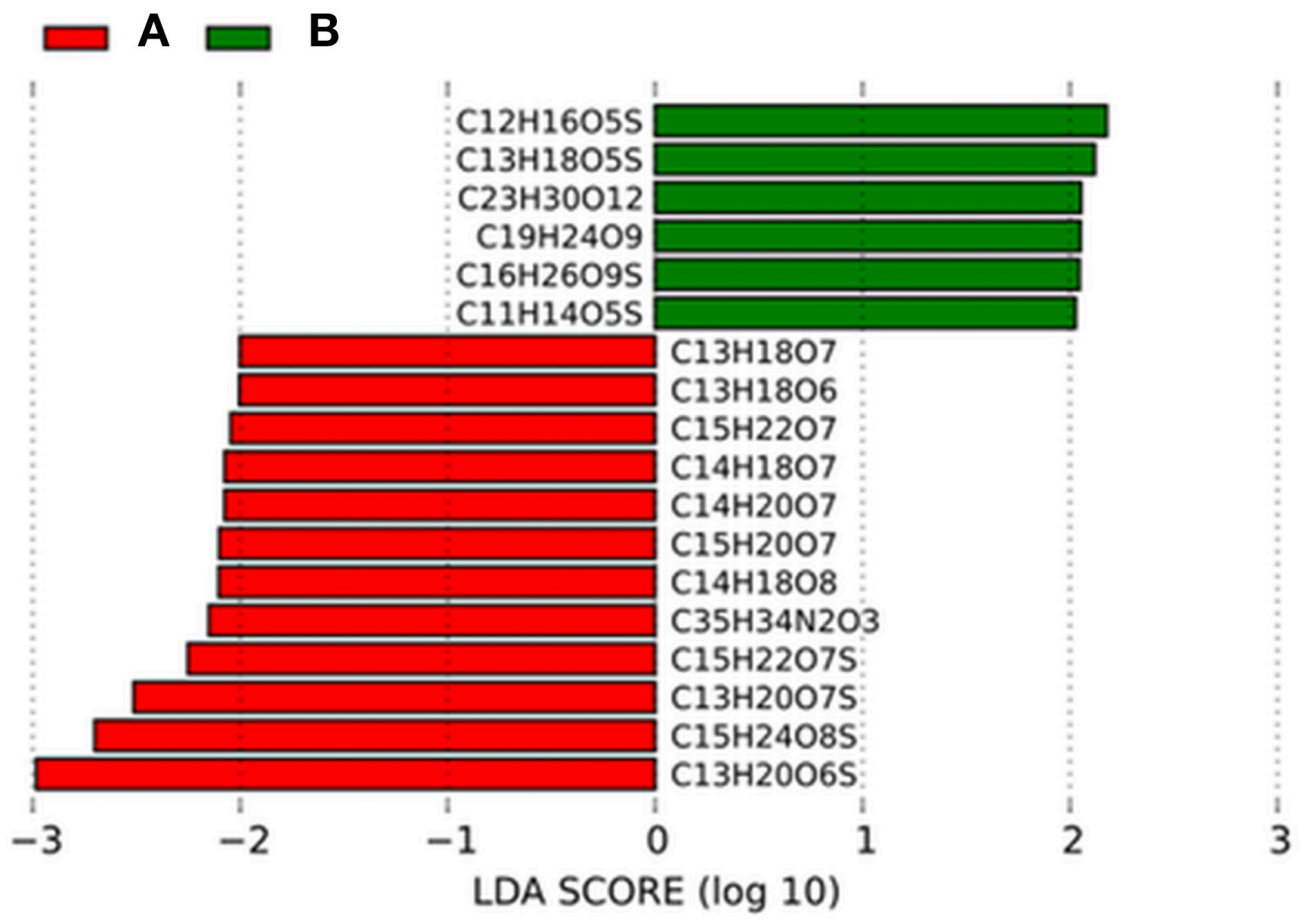
**Linear discriminant effect size analysis (LEfSe) results on DOM molecular formulae**. Histograms of linear discriminant analysis (LDA) scores computed for DOM molecules, differently abundant in group A and B. LDA scores can be interpreted as the degree of consistent difference in relative abundance between the two groups. The histogram thus identifies which DOM formulae among all those detected as statistically different explain the greatest difference between group A and B.

LEfSe analysis identified few molecules that were significantly contributing to the differences in DOM composition between the two groups (Figure 5). Those molecules belonged mainly to the category of highly unsaturated compounds (AI_mod_ ≤ 0.5 and H/C < 1.5) that increased in relative abundance from 80.9% in group A to 82.1% in group B and unsaturated aliphatics (2.0 > H/C ≥ 1.5) that decreased slightly from group A (8.9%) compared to group B (8.1%).

### Linking bacterial communities with molecular DOM composition

**Figure 6:**
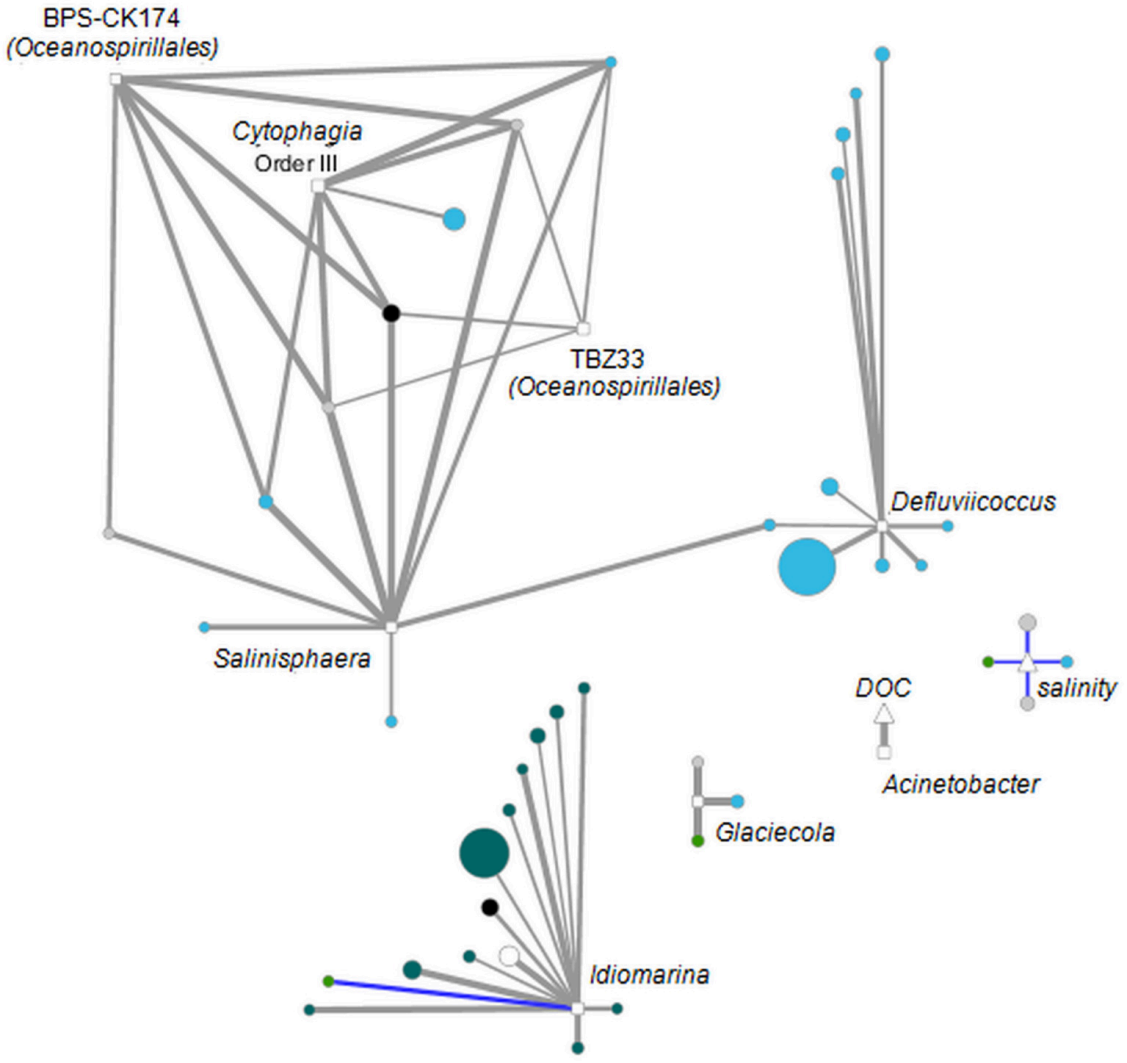
**Interaction network analysis of OTUs (squares), DOM molecules (circles) and environmental parameters (triangles) that were significantly correlated (***p*** < 0.05) with ***R*** ≥ 0.9 or ***R*** ≤ −0.9 (Table 1)**. Positive correlations are indicated in gray, negative correlations in blue. Line width is set proportional to correlation strength. Average OTU relative abundance and DOM molecules abundances are set proportional to node size. Nodes are colored according to DOM category. Blue, unsaturated aliphatics; Petrol, saturated fatty acids; Gray, polyphenols; Black, peptides; Green, highly unsaturated compounds; White, unspecified.

Although similar patterns in bacterial community structure and molecular DOM composition have been observed via PCoA, statistical analysis (PRIMER-E; RELATE subroutine, data not shown) failed to confirm co-variation of both; i.e., among-sample relationships within the sequence data set differed from that within the DOM data set. Nevertheless, strong correlations between single OTUs and DOM molecules were detected. The majority of significant correlations (*p* < 0.05) exhibited correlation coefficients in the range of 0.5–0.6 (Table 1). As the coefficient increased, the number of significant correlations decreased to 56 with *R* ≥ 0.9 of which 51 were exhibited between DOM molecules and OTUs. These strong correlations were formed between only seven OTUs and 36 DOM molecules (Figure 6, Figure S2). Five OTUs belonged to the *Gammaproteobacteria*, one OTU to the *Alphaproteobacteria* and one to the *Cytophagia*. Most of the DOM compounds belonged to unsaturated aliphatics (2.0 > H/C ≥ 1.5) or saturated fatty acids (H/C ≥ 2.0 or O/C ≥ 0.9). A group of seven distinct DOM compounds exhibited strong correlations (*R* ≥ 0.9) with more than one OTU, whereas the remaining DOM compounds were correlated with either, *Defluviicoccus, Idiomarina, or Glaciecola*. Strong correlations of *Defluviicoccus* were restricted to unsaturated aliphatics, whereas strong correlations of *Idiomarina* occurred almost exclusively with saturated fatty acids. All OTUs exhibiting strong correlations also belonged to the ones contributing most to the differences between groups A and B as revealed by LEfSe analyses (Figures 3, 5).

**Table 1:** **Pivot-table for spearman rank order correlations between DOM molecules and environmental parameters, DOM molecules and OTUs, and OTUs and environmental parameters**.

**Coefficient**	**<0.5**	**0.5 – 0.6**	**0.6 – 0.7**	**0.7 – 0.8**	**0.8 – 0.9**	**0.9 – 1**	**Total**
DOM and Env	789 (14.3)	2796 (50.5)	1296 (23.4)	517 (9.3)	130 (2.3)	5 (0.1)	5533
neg	462	1603	769	271	96	5	
pos	327	1193	527	246	34	0	
OTUs and DOM	4248 (16.6)	14,910 (58.2)	5087 (19.9)	1126 (4.4)	183 (0.7)	51 (0.2)	25,605
neg	2262	7912	2522	466	32	1	
pos	1986	6998	2565	660	151	50	
OTUs and Env	17 (10.1)	84 (50)	42 (25)	19 (11.3)	6 (3.6)	0 (0)	168
neg	7	34	18	12	3	0	
pos	10	50	24	7	3	0	
Sum	5054	17,790	6425	1662	319	56	31,306

*Correlations are sorted according to correlation strength. Numbers of correlations are given. Numbers in brackets refer to percentage of correlations on total correlations within the corresponding group. Env, environmental parameter; neg, negative correlations; pos, positive correlations*.

## Discussion

### Impact of environmental conditions on BCC

Salinity dynamics at Helgoland Roads are mainly controlled by hydrological and meteorological forces and by the huge runoff from the rivers Elbe and Weser (Atlas and Bartha, 1987). Long-term studies of oceanographic environmental parameters at Helgoland Roads reported mean annual salinities ranging between 31 and 33 (Raabe and Wiltshire, 2009). Transport of central North Sea water toward Helgoland Roads results in high salinities, whereas coastal water influx is related to lower salinities (Wiltshire et al., 2010). Low salinity events at Helgoland Roads presumably occur during winter months, especially in February, when the Elbe discharge is particularly high (Raabe and Wiltshire, 2009). During this study conducted in August 2012, the recorded salinity values exhibited a salinity shift of ~1.5 within 4 days which is exceptional during usually more stable hydrographic conditions in summer and points to a strong short-term influence of coastal water masses.

**Figure 7:**
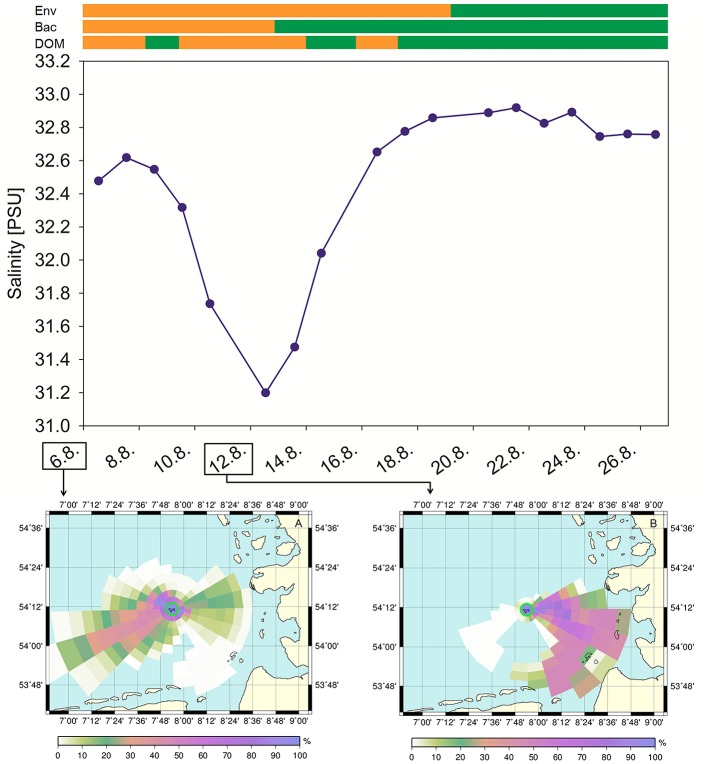
**Histories of water bodies observed at Helgoland Roads**. Based on pre-calculated near surface current velocities from the hydrodynamic model BSHcmod, 500 passive tracer particles were tracked backward in time. Referring to a cobweb like grid structure, the color scale reflects the percentage of particle trajectories that touched a given grid cell at any time within the past 3 weeks. Bars on top depict group formation for environmental parameters (Env), bacterial communities (Bac) and DOM. Orange, group A; Green, group B.

The intermittent change of water masses during our study is confirmed by the results of tracer particle backtracking. Trajectories were simulated with PELETS-2D (Callies et al., 2011) based on pre-calculated near surface current velocity fields from the hydrodynamic model BSHcmod (Dick et al., 2001) operated by the Federal Maritime and Hydrographic Agency of Germany (Bundesamt für Seeschifffahrt und Hydrographie, BSH). Model results (Figure 7) help delineate regions of origin by analyzing the percentages of particle trajectories that crossed certain grid cells over the previous 3 weeks. We organized grid cells in a cobweb like structure centered at Helgoland Roads in order to take account of uncertainty increasing with distance. Figure 7 clearly documents an event with strong advection from the inner German Bight (near the Elbe estuary) toward Helgoland during the period around the 12th of August (Figure 7, right bottom panel) when salinity values at Helgoland were found to drop substantially. According to model simulations, this inshore origin of water masses did not exist (or was at least much less pronounced) at both the beginning and the end of the sampling period.

Statistical analysis on 16S rRNA tag data suggested a separation of samples into two groups that is particularly influenced by temperature, TDN, O_2_, ${\text{PO}}_{4}^{3-}$, and DOC. Multicollinearity of parameters describing ecological conditions might lead to biased interpretation of linear regression models (Graham, 2003). Correlation analysis confirmed multicollinearity of salinity with O_2_ and DOC, which hints at a shared contribution of these parameters. In conjunction with the above mentioned coastal water inflow this leads to the assumption that salinity can be interpreted as a proxy for different water bodies with differing environmental conditions.

TDN is composed of dissolved inorganic nitrogen (DIN) and dissolved organic nitrogen (DON). DON comprises a large pool of fixed nitrogen in most aquatic systems, accounting for as much as 40–70% of the TDN pool in surface seawater (Bronk, 2002). It consists of labile, rapidly overturning proteins, amino polysaccharides, urea, and nucleic acids, but also includes more refractory compounds like humic acids (e.g., Bronk et al., 2007), most of which derive from primary producers but also from bacterial cell wall material (McCarthy et al., 1998). TDN concentrations measured during this study increased toward the end of the sampling period and reached highest concentrations shortly after the Chl *a* maximum. Thus, the increase in TDN concentration might reflect the permanent release of DOM by phytoplankton during its growth phase and additional release due to grazing or viral lysis that may affect the termination of the bloom (Beare et al., 2002; Wiltshire et al., 2010). However, it has to be noticed that DON concentration was not measured thus, this interpretation is speculative and needs to be evidenced.

In general, the bacterial community observed in our study was dominated by *Flavobacteriia, Alpha-*, and *Gammaproteobacteria*. These classes have been consistently found to dominate bloom-associated bacterial communities as reviewed by Buchan et al. (2014). Also, the most abundant OTUs found during this study are common members of the North Sea bacterial community during phytoplankton blooms (e.g., Giebel et al., 2011; Teeling et al., 2012; Wemheuer et al., 2014). Comparison of bacterial communities of group A, with low average TDN concentration (14.61 μM) and group B exhibiting higher TDN concentrations (19.98 μM) revealed that group B is characterized by higher relative abundance of *Flavobacteriia* (*Formosa, Fluviicola*, NS2b, *Crocinitomix*, and NS7 marine group) which are well known to be active in biopolymer degradation and reacting to phytoplankton blooms (e.g., Teeling et al., 2012; Buchan et al., 2014; Lucas et al., 2015). *Formosa* for instance has been found to be among the first taxa responding to a phytoplankton bloom and dominating the bacterial community at Helgoland Roads (Teeling et al., 2012). In contrast, some *Alpha-* and *Gammaproteobacteria* related OTUs decreased in relative abundance from group A to B. For instance *Defluviicoccus* (*Alphaproteobacteria*) exhibited slightly higher relative abundances in Group A (0.3%) than in group B (0.1%). *Defluviicoccus* is not a typical representative of marine *Alphaproteobacteria* and is usually found in wastewater treatment plants (Nobu et al., 2014), which might explain the occurrence of this OTU more frequently in coastal waters than in marine oceanic environments. Another OTU, *Idiomarina* (*Gammaproteobacteria*) showed increased relative abundances in group A (3.1%) compared to group B (0.3%). Also *Idiomarina* spp. is not a typical representative of the marine waters around Helgoland. It has been isolated from various marine environments such as deep-sea waters, hydrothermal vents, sediments, and reef building corals (e.g., Ivanova et al., 2000; Donachie et al., 2003; Chen et al., 2012; Zhang et al., 2012), but also from estuarine environments like Baltic Sea surface water (Brettar et al., 2003). The increased occurrence of these two OTUs at Helgoland Roads during the first half of the sampling period (group A), leads to the hypothesis that OTUs that are commonly associated with coastal marine environments have been passively transported to Helgoland Roads with the above mentioned coastal water inflow. Thus, *Defluviicoccus* and *Idiomarina* reflect the short-term impact of coastal water inflows on the BCC at Helgoland Roads.

### Impact of environmental conditions on molecular DOM composition

Variability in the DOM composition was mainly driven by salinity and temperature as revealed by DistLM (Figure 2E). The molecules that were positively correlated with salinity exhibited higher H/C ratios and were clearly separated from molecules that were negatively correlated with salinity and showed lower H/C values (Figure 4A). In general, marine DOM has higher H/C ratios, is more aliphatic and contains higher proportions of carbohydrates, amino acids, and lipids, whereas terrestrial DOM is more aromatic, contains carboxyl, and hydroxyl functionalities and has lower H/C ratios (Sleighter and Hatcher, 2008; Medeiros et al., 2015; Seidel et al., 2015). Similar observations by Kim et al. (2003) and Koch et al. (2005) support the assumption that the molecules positively correlated with salinity are associated with marine DOM and those negatively correlated are associated with terrigenous DOM. This interpretation is also supported by our findings that on average unsaturated aliphatics (2.0 > H/C ≥ 1.5) were most abundant during the coastal water inflow. Thus, we conclude that differences between the observed groups in this study can be partly explained by different water masses and thus origins of DOM.

Furthermore, we found that molecules negatively correlated with temperature had higher H/C ratios than positively correlated molecules (Figure 4B). Higher H/C ratios indicate higher saturation which is characteristic for compounds that are rapidly degradable, e.g., fatty acids and proteinaceuos material. As temperature increased toward the end of the sampling period, molecules with high H/C ratio decreased. This observation could be explained by a scenario were microbial activity had increased with rising temperatures and as response to enhanced organic matter supply released by phytoplankton. Due to enhanced metabolism, the microbial community may have consumed more DOM (Pomeroy and Wiebe, 2001), which could have preferentially diminished the pool of labile DOM and thus, diminished the amount of molecules with high H/C ratio. The notion of enhanced microbial activity is also supported by the H/C ratios of molecules correlated with DOC. The simultaneous increase of compounds with low H/C ratios and DOC concentration (reflected in the positive correlation of these compounds with DOC) support the scenario of preferential consumption of rapidly degradable compounds with high H/C ratios leading to an increasing relative abundance of molecules with low H/C ratios.

### Relation between bacterial community and molecular DOM composition

Linkage of bacterial relative abundances with DOM data revealed evidence for dependency of specific OTUs on particular DOM molecules. Especially *Gammaproteobacteria* showed strong positive correlations (*R* ≥ 0.9) with unsaturated aliphatics and saturated fatty acids (Figure 6), all of which showed decreased relative abundances in group B compared to group A. *Gammaproteobacteria* are known to be typical marine bacteria, thus one would expect increasing relative abundances in group B, which exhibits higher salinity than group A, which was strongly influenced by a coastal freshwater inflow. A possible explanation for the observed predominance of *Bacteroidetes* in group B might be the onset of the summer phytoplankton bloom that occurred in group B and might have supported enhanced growth of *Bacteroidetes* by providing complex organic compounds. Thus *Gammaproteobacteria* relative abundances might have decreased due to increasing *Bacteroidetes* abundances.

Network analyses revealed few DOM compounds that were highly correlated (*R* ≥ 0.9) with different bacteria taxa and thus seem to serve as a general substrate. On the other hand we observed strong correlations of *Defluviicoccus* (*Alphaproteobacteria*) with unsaturated aliphatics as the only substrate category (Figure 6), which might indicate that *Defluviicoccus* specialized on selected DOM compounds that are not as intensively consumed by other taxa and which might be important for defining its ecological niche. As already mentioned *Defluviicoccus* spp. is typically found in wastewater treatment plants (e.g., Nobu et al., 2014), and capable of taking up a narrow range of substrates such as acetate, propionate, pyruvate, and glucose (Burow et al., 2007), which supports our findings of strong correlations with unsaturated aliphatics. Another example for specialization on specific substrate classes is *Idiomarina* (*Gammaproteobacteria*), which is almost exclusively strongly correlated with specific saturated fatty acids (Figure 6). The notion of specific bacterial taxa specializing on selected DOM molecules is supported by previous studies that also suggested coordinated resource partitioning by bacterial specialists leading to a defined temporal succession of bacterial taxa (e.g., McCarren et al., 2010; Teeling et al., 2012). Nevertheless, as discussed above we hypothesize that relative abundances of *Defluviicoccus* and *Idiomarina* at Helgoland Roads are influenced by the observed coastal water inflow. The same might be true for the different DOM compound classes. Thus, it has to be further analyzed whether the simultaneously decreasing relative abundances of unsaturated aliphatics and *Defluviicoccus* and saturated fatty acids and *Idiomarina* represent real dependencies, or just reflect a simple co-occurrence in different water bodies. However, so far studies that dealt with resource partitioning by bacterial specialists analyzed the transcriptional responses of microbial communities to high-molecular-weight DOM amendment or enhanced substrate supply by phytoplankton blooms. Here, we demonstrate the possibility to link single bacterial taxa to specific DOM formulae rather than just molecule categories. Even though FT-ICR-MS has, as any analytical technique, a defined analytical window, it is an important step to further unravel the specific microorganisms and metabolic pathways responsible for the degradation and transformation of DOM in the oceans.

Although we were able to relate single OTUs with specific DOM molecules, a direct general relationship between bacterial community structure and DOM composition could not be demonstrated. One possible explanation might be that freshly produced labile DOM that is accessible for microorganisms is rapidly turned over by the bacterial community as shown in several studies (Kirchman et al., 1991; Amon and Benner, 1996; Weiss and Simon, 1999). Thus, the pool of labile DOM compounds that could have a significant influence on bacterial community structure might not be detectable with the methods applied in this study. An instantaneous degradation of introduced fresh DOM by bacterioplankton is also proposed for arctic fjords (Svalbard), which results in a predominance of the prevailing semi-refractory and refractory DOM pool Osterholz et al. (2014).

Furthermore, methodological limitations could have led to lacking evidence of a relation between the bacterial community structure and DOM composition. A previous study by Flerus et al. (2012) suggested that colloidal material and low molecular weight DOM (<250 Da) can be lost during solid phase extraction (SPE) as used in this study. Thus, labile DOM that might have an influence on bacterial community structure, escapes the analytical window. The low extraction efficiencies observed during our study might indicate that a substantial fraction of DOM was not extracted. Although the efficiencies were in the range described for marine samples (Dittmar et al., 2008), they were considerably lower than in more recent studies (Rossel et al., 2013; Osterholz et al., 2014). Despite these methodological limitations inherent to any analytical method, we identified significant variations in DOM composition and successfully linked them to environmental conditions and BCC.

To our knowledge this is the first time that dynamics of BCC and molecular DOM composition have been documented on high temporal and analytical resolution. We conclude that the bacterial community is highly influenced by freshly produced, labile DOM pulses as derived from phytoplankton blooms. Rapid transformation of labile DOM might lead to selective relative enrichment of more refractory DOM and thus hamper the detection of interdependencies between bacterial community structure and DOM composition. High resolution techniques like 16S rRNA tag sequencing and FT-ICR-MS provide substantial information on substrate generalists and specialists and may help to predict changes in BCC. To further unravel the relationship between bacteria and molecular DOM composition it has to be considered that metabolic capabilities are not restricted to specific phylogenetic groups. Thus, for future analyses we suggest combining FT-ICR-MS analyses of DOM with functional approaches of bacterial communities rather than phylogenetic description.

## Author contributions

GG, AW, TD, and JN conceived the study. IK and JN performed sampling. IK performed DOM-analysis. JL performed bacterial community and statistical analysis. UC performed tracer particle backtracking analysis. KW provided environmental parameters. All authors contributed to data interpretation. JL and IK wrote the manuscript with significant input from all co-authors.

### Conflict of interest statement

The authors declare that the research was conducted in the absence of any commercial or financial relationships that could be construed as a potential conflict of interest. The reviewer QZ and handling Editor declared their shared affiliation, and the handling Editor states that the process nevertheless met the standards of a fair and objective review.
