# Supplementary material for: Short-Term Dynamics of North Sea Bacterioplankton-Dissolved Organic Matter Coherence on Molecular Level
Source: Front Microbiol. 2016 Mar 15;7:321. doi: 10.3389/fmicb.2016.00321 (PMC4791370; doi:10.3389/fmicb.2016.00321)
Supplement: Supplementary file 1 [file Presentation1.PDF]

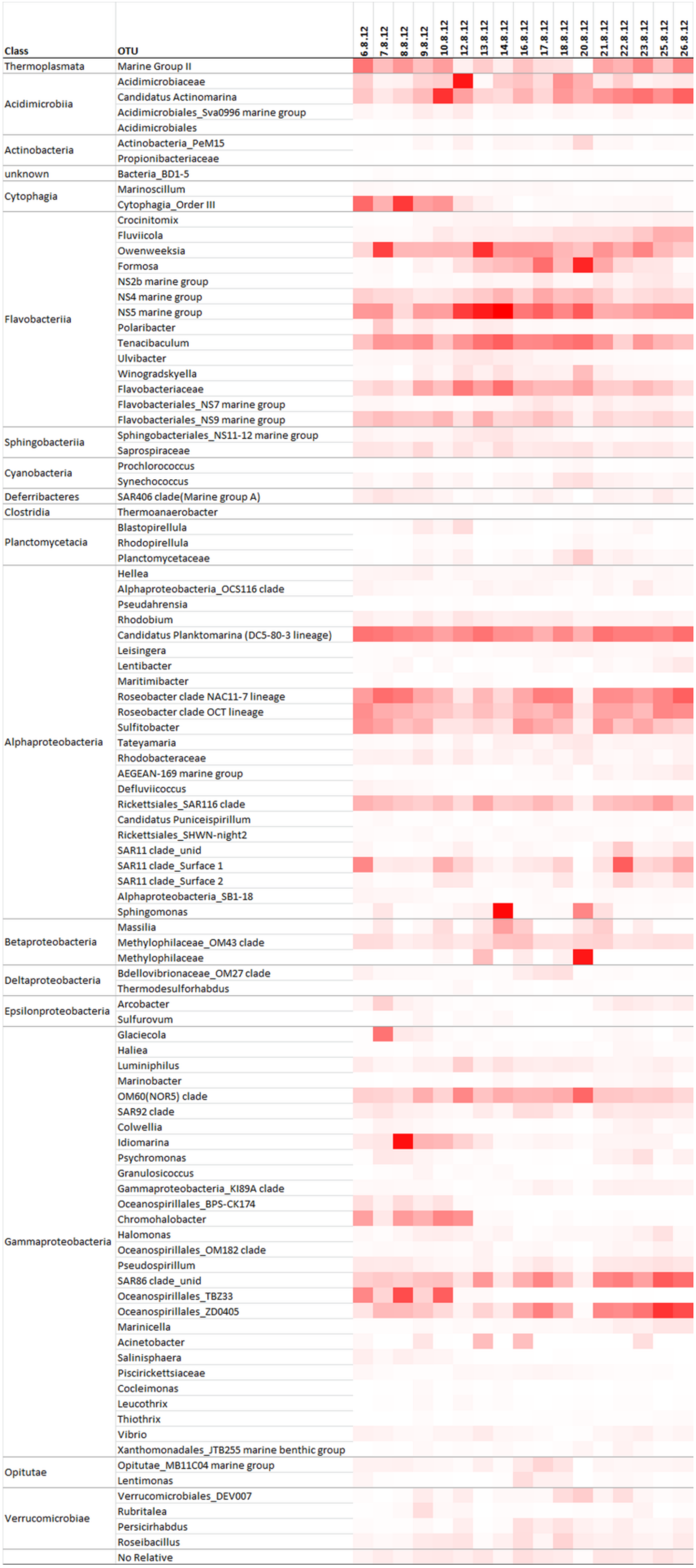

Figure S1:

Relative abundances of the 98 different OTUs over the course of the sampling period. The colour scale refers to the contribution of each OTU to the total community given in percentage. Dark red reflects high percentages, light red to white reflects low percentages.

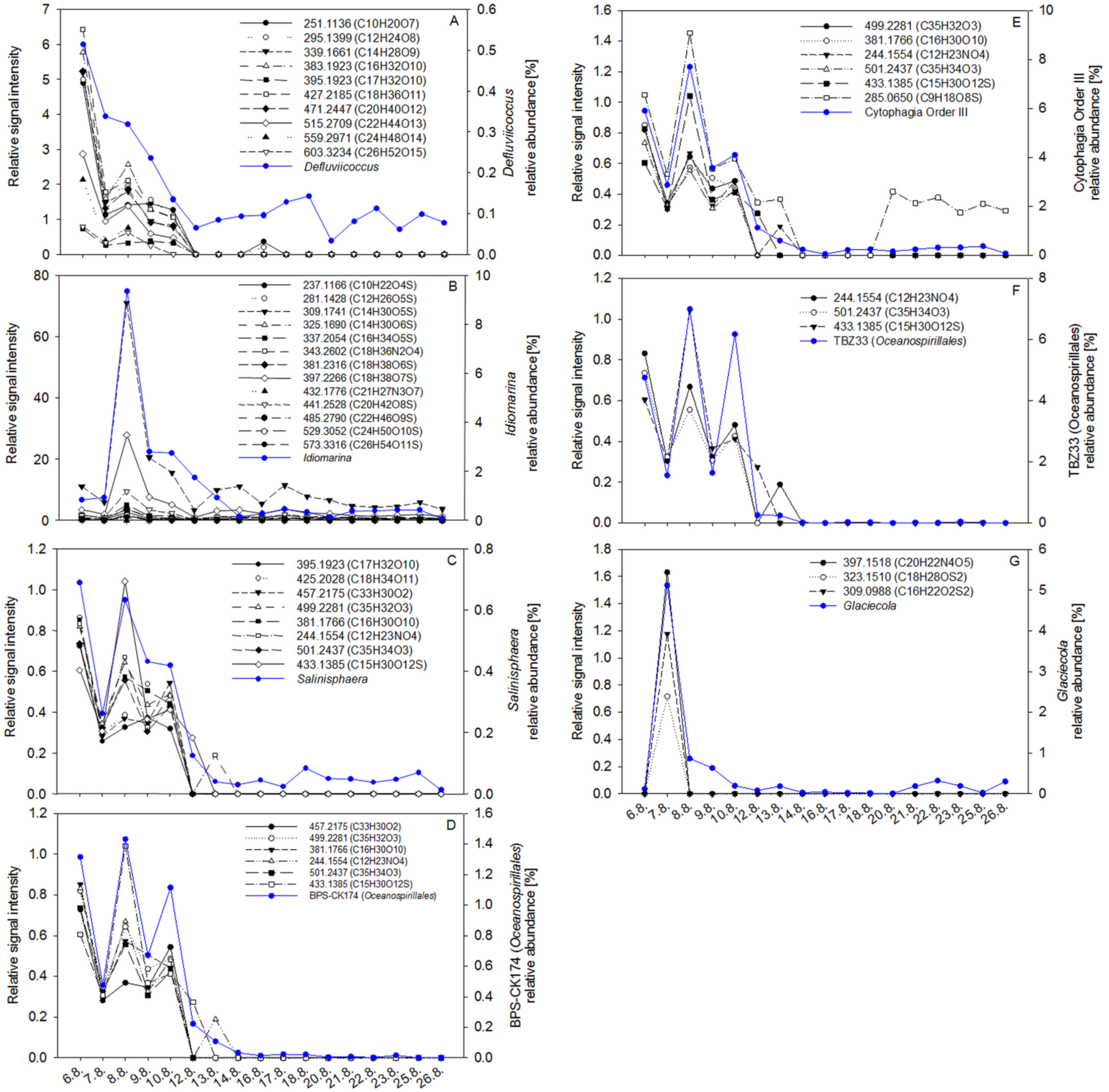

Figure S2: Relationship of specific bacterial OTUs with particular DOM molecules. Relative signal intensities and relative abundances of DOM molecules and OTUs that were highly correlated (R>0.9) are depicted for the whole sampling period. The curves of the OTUs and the correlated DOM molecules exhibit similar behavior during the time course. Thus artificial correlations that might occur due to the size of the data set can be excluded.
